# Effective DNA/RNA Co-Extraction for Analysis of MicroRNAs, mRNAs, and Genomic DNA from Formalin-Fixed Paraffin-Embedded Specimens

**DOI:** 10.1371/journal.pone.0034683

**Published:** 2012-04-13

**Authors:** Adam Kotorashvili, Andrew Ramnauth, Christina Liu, Juan Lin, Kenny Ye, Ryung Kim, Rachel Hazan, Thomas Rohan, Susan Fineberg, Olivier Loudig

**Affiliations:** 1 Department of Epidemiology and Population Health, Albert Einstein College of Medicine of Yeshiva University, Bronx, New York, United States of America; 2 Department of Pathology, Albert Einstein College of Medicine of Yeshiva University, Bronx , New York, United States of America; University of Hong Kong, Hong Kong

## Abstract

**Background:**

Retrospective studies of archived human specimens, with known clinical follow-up, are used to identify predictive and prognostic molecular markers of disease. Due to biochemical differences, however, formalin-fixed paraffin-embedded (FFPE) DNA and RNA have generally been extracted separately from either different tissue sections or from the same section by dividing the digested tissue. The former limits accurate correlation whilst the latter is impractical when utilizing rare or limited archived specimens.

**Principal Findings:**

For effective recovery of genomic DNA and total RNA from a single FFPE specimen, without splitting the proteinase-K digested tissue solution, we optimized a co-extraction method by using TRIzol and purifying DNA from the lower aqueous and RNA from the upper organic phases. Using a series of seven different archived specimens, we evaluated the total amounts of genomic DNA and total RNA recovered by our TRIzol-based co-extraction method and compared our results with those from two commercial kits, the Qiagen AllPrep DNA/RNA FFPE kit, for co-extraction, and the Ambion RecoverAll™ Total Nucleic Acid Isolation kit, for separate extraction of FFPE-DNA and -RNA. Then, to accurately assess the quality of DNA and RNA co-extracted from a single FFPE specimen, we used qRT-PCR, gene expression profiling and methylation assays to analyze microRNAs, mRNAs, and genomic DNA recovered from matched fresh and FFPE MCF10A cells. These experiments show that the TRIzol-based co-extraction method provides larger amounts of FFPE-DNA and –RNA than the two other methods, and particularly provides higher quality microRNAs and genomic DNA for subsequent molecular analyses.

**Significance:**

We determined that co-extraction of genomic DNA and total RNA from a single FFPE specimen is an effective recovery approach to obtain high-quality material for parallel molecular and high-throughput analyses. Our optimized approach provides the option of collecting DNA, which would otherwise be discarded or degraded, for additional or subsequent studies.

## Introduction

Archived human specimens, with known clinical follow-up, represent a valuable resource, particularly for retrospective molecular studies and identification of biological markers that might be useful for risk prediction of disease or prognosis [1].

Recent studies have demonstrated that nucleic acids recovered from archived specimens are suitable for a variety of downstream genetic (genomic and transcriptomic) and epigenetic analyses [1]. Genomic DNA recovered from archived specimens, while degraded, can be analyzed by polymerase chain reaction (PCR) [2], [3], array comparative genomic hybridization (CGH) [4], massively parallel sequencing [5], and methylation assays [6]–[8]. Contrastingly, messenger RNA molecules recovered from formalin-fixed paraffin-embedded (FFPE) specimens display a large extent of degradation, and thus many studies have aimed at demonstrating their suitability for molecular analyses and specific protocols have been established for quantitative reverse transcription PCR (qRT-PCR) [2], [9], high-throughput gene expression [1], [10]–[12], and even massive parallel sequencing [13], [14]. Interestingly, microRNAs, due to their small size, remain intact throughout the processes of formalin-fixation and RNA extraction, and they can be reliably studied in FFPE specimens [15], [16].

Protocols for genomic DNA or total RNA extractions, from FFPE specimens, have been well documented and made available as reliable commercial kits [17], [18]. In general, tissue sections are deparaffinized in a non-polar solvent: xylene, Hemo-D (d-limonene), or citrisolv and then subjected to proteinase-K digestion, usually short (15 minutes to overnight) for RNA, to minimize degradation, but extended (for up to 48 h) for DNA isolation [1]. To increase DNA purity, exposure to high-temperature (95–98°C), in an alkaline buffer, has been shown to allow removal of DNA/protein cross-links, a denaturing step however that cannot be used during RNA isolation [19]–[22]. To avoid cross-contamination between these two types of nucleic acids, an RNase or DNAse treatment for DNA or RNA purification, respectively, is added prior to either a solvent separation (TRIzol, phenol/chloroform) or a silica-based column purification [11], [18]. To increase RNA quality, a final step consists of heat-treatment at 70°c for up to 60 minutes, in a Tris-EDTA (1×TE) or citrate-based buffer, to remove chemical modifications (methylol groups) acquired during formalin-fixation [23], [24]. Based on these different biochemical requirements, DNA and RNA have routinely been extracted separately.

The recovery of genomic DNA and total RNA from the same specimen has the advantage of providing matched nucleic acid fractions, from the same cells, which is extremely valuable for validations as well as for integrative studies. Maximizing DNA and RNA retrieval from a single specimen might also be very useful when using tissues that are of limited availability.

In this study, we sought to determine if genomic DNA and total RNA could be effectively co-extracted from archived specimens within a single reaction. Therefore we optimized a co-extraction method using TRIzol, which is the most trusted reagent for total RNA extraction from fresh tissues, because it allows DNA/RNA phase separation and recovery from fresh tissues [25]. Then, using a series of seven human archived specimens, we quantitatively compared our optimized approach to two commercial kits designed for either simultaneous (Qiagen AllPrep DNA/RNA FFPE kit) or separate (Ambion RecoverAll™ Total Nucleic Acid Isolation kit) DNA or RNA extractions [26]. Finally, using material recovered from matched fresh and one month-old FFPE MCF10A cells, we simultaneously assessed the quality of mRNA by quantitative RT-PCR and global gene expression using the whole-genome cDNA-mediated Annealing, Selection, Extension and Ligation (WG-DASL) assay from Illumina, microRNAs by qRT-PCR and expression profiling, and genomic DNA by methylation assays.

## Materials and Methods

### Specimens

Formalin-fixed paraffin-embedded (FFPE) specimens were obtained from Dr. Susan Fineberg at the Montefiore Medical Center (MMC), Bronx, NY. In accordance with OHRP Guidance on research involving coded private information or biological specimens, this study did not meet the definition of human subject research as defined by 45 CFR 46.102(f), as data/specimens were not collected specifically for the proposed research project and the data/specimens received by Dr. Loudig did not contain a code derived from individual personal information. Thus, experiments using these tissue blocks did not require further monitoring from the Albert Einstein College of Medicine Institutional Review Board (IRB), which also oversees MMC. Electrophoretic analysis and methylation analyses of genomic DNA from older FFPE benign breast tissue samples (8, 13, 20, 25, 27 and 31 year-old) were performed with specimens obtained from Kaiser Permanente Northwest, after approval of a pilot study entitled “Gene Methylation and Oxidative Stress in the Etiology of breast Cancer” from the ethical board, which was supervised by Dr. Thomas Rohan. IRB approval for this study was obtained from the Albert Einstein College of Medicine Institutional Review Board and from the Kaiser Permanente Northwest Institutional Review Board (Portland), which waived the requirement to obtain informed consent prior to use of these specimens. The tissue blocks were cut on a standard microtome (Leica-microsystems) to generate successive 10 µm sections. Fresh mouse tissues were recovered from dead animals after they had been sacrificed and analyzed in the laboratory of Dr. Rachel Hazan at the Albert Einstein College of Medicine. The animal use protocol was reviewed and approved by the Animal Institute Committee (AIC) of Albert Einstein College of Medicine, the institution's animal care and use committee, on 11/06/08. AIC approved the protocol for a period of 3 years from the approval date. The approved Animal Welfare Assurance (A3312-01) is on file with the Office for Laboratory Animal Welfare. Albert Einstein College of Medicine has been fully accredited by the Association for the Assessment and Accreditation of Laboratory Animal Care (AAALAC) since February 22, 1983. This protocol was renewed for a period of 3 additional years on 11/06/11. The tissues were processed with TRIzol in the laboratory of Dr. Rachel Hazan.

### Methods for RNA and DNA Extraction from Fresh Tissues and Cells

Genomic DNA from fresh tissue (mouse) and cells (human MCF10A) was extracted using phenol/Chloroform method or TRIzol, following manufacturer's instructions (Invitrogen, CA, USA).

### Optimized Method for Co-Extraction of RNA and DNA from FFPE Tissue

Using the TRIzol-based method described in Loudig *et al.* 2007, total FFPE-RNA was obtained from the upper aqueous phase of TRIzol, and genomic FFPE-DNA from the lower organic phase of TRIzol [11]. FFPE-DNA was precipitated by addition of 1200 µl of ethanol and 20 µl of sodium acetate (NaAc), incubation at room temperature for 3 minutes, and centrifugation at 16,000RPM for 30 min at 4°C. The DNA pellet was washed with 100% ethanol, air-dried 50°C, re-suspended in 180 µl ATL buffer from the DNA FFPE kit (Qiagen, CA, USA), and subjected to proteinase K (pK) digestion for 48 hours at 56°C (20 µl of pK (30 mg/ml) at start and at 24 h). After 48 h, the solution was incubated at 90°C for 1 h, 200 µl of AL buffer (Qiagen DNA FFPE kit) and 200 µl of 100% ethanol were added to the solution, which was vortexed and transferred to a MinElute column. The column was spun at 8,000RPM for 1 min and washed with 500 µl AW1 and AW2 buffers, successively. The column was dried by centrifugation at 14,000RPM for 3 minutes and the DNA was eluted by addition of 20 µl of 1×TE buffer and centrifugation. The DNA was quantified on a NanoDrop ND-1000 spectrophotometer and analyzed on 1% agarose gel prior to methylation assays.

### Commercial Kits for Extraction of RNA and DNA from FFPE Tissue

For extraction of FFPE-DNA alone, we used the QIAamp DNA FFPE kit (Qiagen, CA, USA) following manufacturer's instructions, using 24 hours proteinase K (pK) digestion. For co-extraction of FFPE-DNA and -RNA we used the Qiagen AllPrep DNA/RNA FFPE kit following manufacturer's instructions. We used the RecoverAll™ Total Nucleic Acid Isolation kit (Ambion, TX, USA) to extract FFPE-DNA or -RNA, and following manufacturers' instructions the pK digested FFPE tissue solution was separated into two halves, with one half subjected to DNase for FFPE-RNA purification, and the other half left at 55°C for 16 hours before RNase treatment and DNA purification. FFPE-RNA alone was extracted with the High-Pure RNA Paraffin kit (Roche, IN, USA) for analysis on the Whole-Genome cDNA-mediated Annealing, Selection, Extension and Ligation (DASL) assay.

### Tissue Culture

Non-tumorigenic breast epithelial MCF10A cells were obtained from Dr. Paraic Kenny at the Albert Einstein College of Medicine and they were cultured in DMEM/F12 (Cellgro, VA, USA), supplemented with 5% horse serum (Invitrogen, CA, USA), hydrocortisone (0.5 µg/ml), mouse epidermal growth factor (EGF; 20 ng/ml), insulin (10 µg/ml), cholera toxin (100 ng/ml, Sigma, MO, USA) at 37°C in a humidified incubator (5% CO2). Fresh and FFPE cells were prepared as described in Loudig *et al.* 2011 [26].

### Microrna Expression Profiling

Total RNA (200 ng) from fresh and FFPE cells was subjected to high-throughput miRNA profiling (1,146 miRNAs) using the Illumina miRNA platform (Illumina, CA, USA) on 12 beadchip arrays, according to manufacturer's instructions, as described in Giricz *et al.* 2011 [16]. Arrays were scanned on a beadarray reader and raw data were obtained using GenomeStudio.

### Messenger RNA Expression Profiling

mRNA expression profiling (24,526 features) was performed with total RNA extracted from fresh and FFPE cells (200 ng) using the Illumina Whole genome cDNA-mediated Annealing, Selection, Extension and Ligation (DASL) assay on 32 beadchip arrays, following manufacturer's instructions and according to Loudig *et al.* 2011 [26]. Beadchip arrays were scanned on a Beadarray Reader (Illumina, CA, USA) and raw data were obtained using GenomeStudio.

### Microrna and Messenger RNA Quantitative RT-PCR Experiments

MicroRNAs miR-10a, miR-196b, miR-135b, miR-32a and miR-21 were quantified from total RNA from fresh and FFPE cells using Taqman® miRNA qRT-PCR (Applied Biosystems, CA, USA) as described in Giricz et al 2011. RNU44 and RNU6B were used as endogenous controls for data normalization as described in Giricz *et al.* 2011 [16]. mRNAs for ESR1, CCND2 and KRT14 were quantified in matched fresh and FFPE RNA using Taqman® gene expression qRT-PCR reagents (Applied Biosystems, CA, USA). Two sets of Taqman® primers for GAPDH were used as endogenous controls for data normalization. Fold-change differences between fresh and FFPE RNA were calculated as described in Loudig *et al.* 2011 [27].

### DNA Methylation Analysis

Methylation was assayed using the procedure described by Thompson *et al.* 2009 [28]. Sodium bisulfite treatment was performed with 100–200 ng of fresh and FFPE-DNA using the EZ DNA methylation direct kit (Zymo Research, CA, USA), following manufacturers' protocol. PCR primers were designed using Methprimer for methylation PCRs [29], verified in-silico using Bisearch [30] and R MasArray statistical package [28], and the UCSC genome browser [31]. PCR amplification was conducted using FastStart High Fidelity DNA polymerase (Roche, IN, USA), for 42 cycles. DNA methylation analysis was performed on PCR products using the MassArray EpiTYPER system (Invitrogen, CA, USA), which uses base-specific cleavage followed by MALDI-TOF mass spectrometry. Each experiment was performed in triplicate and analyzed on the MassArray Statistical package for the R environment [28].

### Statistical Analysis

For FFPE-RNA recovered by four different methods, TRI, QDR, AMB, and Roche, gene expression profiles were measured by WG-DASL assay. Raw expression intensities of mRNAs were normalized by quantile normalization method implemented in GenomeStudio [32]. For each of the four methods, the normalized intensities of three replicates were averaged, and the Pearson rank correlation coefficients between averaged FFPE and each of the three fresh samples were computed. The MicroRNA expression profiles were analyzed and compared in the same fashion between three methods (TRI, QDR, AMB), by computing Pearson correlation coefficient between FFPE and fresh MCF10A cells.

## Results

### Optimization of FFPE-DNA Extraction from the Lower Phase of TRIzol

**Figure 1:**
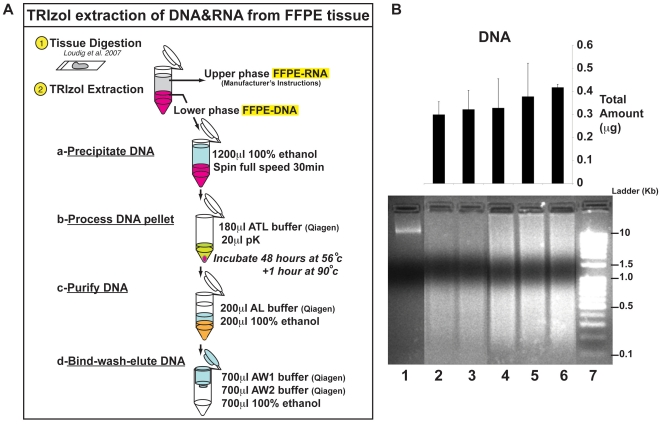
Optimized TRIzol extraction of DNA from archived specimens. (A) Schematic representation of DNA recovery from the lower phase of TRIzol (upper phase yields RNA). In step 1 (yellow bullet), tissue digestion is performed following the procedure described in Loudig *et al.* 2007. In step 2 (yellow bullet), using TRIzol RNA and DNA are separated into the upper and lower phases, respectively. The DNA is recovered from the lower phase, using our optimized approach described in the materials and methods. The four steps describing optimization of DNA recovery from the lower phase of TRIzol include: a. Precipitate DNA; b. Process DNA pellet (using reagents from Qiagen DNA FFPE kit for steps b to d); c. Purify DNA; d. Bind, wash, and elute DNA. (B) Analysis of DNA from FFPE tissue recovered from the lower phase of TRIzol. The upper panel shows the histogram of DNA recovery. The lower panel shows a 1.5% agarose gel electrophoresis image of fresh DNA recovered from a TRIzol treatment lower phase (lane 1), FFPE DNA recovered from a TRIzol lower phase (lanes 2–6), and the size ladder (lane 7). For DNA, precipitation was tested for 600 µl (lane 2 and lane 4), 1000 µl (lane 3 and lane 5), and 1200 µl of Ethanol (lane 6). Proteinase K (PK) treatment was performed for 24 (lanes 2–3) or 48 hours (lanes 4–6). Electrophoresis reveals integrity of the extracted DNA samples. The histogram and agarose gel show that precipitation with a combination of 1200 µl ethanol and 48 hours of PK treatment gives the best quality and quantity of DNA. 500 ng of DNA was loaded per well of the gel.

For fresh tissue, DNA and RNA can be simultaneously but separately extracted from the lower organic and the upper aqueous phase of TRIzol, respectively (Figure S1; see DNA/RNA from fresh mouse brain, muscle, heart and liver tissues). Considering that our optimized RNA extraction method for FFPE tissues [12] uses TRIzol as the final chaotropic reagent, we sought to determine if FFPE-DNA could be precipitated from the lower aqueous phase of TRIzol. While an FFPE-DNA pellet was observable and DNA readable on a NanoDrop ND-1000, it could not be observed on an agarose gel and did not produce PCR amplicons (data not shown). We hypothesized that 45 minutes of proteinase-K (pK) treatment, designed for optimal FFPE-RNA recovery, was insufficient for removing FFPE-DNA/protein cross-linkages and thus we subjected the DNA pellet to additional pK treatment and purified the FFPE-DNA using the Qiagen QIAamp DNA FFPE kit (Fig. 1A). This approach provided consistent yields with observable FFPE-DNA (Fig. 1B).

### DNA and RNA Extraction from Archived Specimens

**Figure 2:**
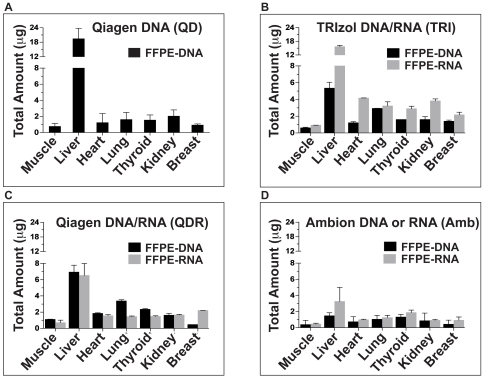
DNA/RNA extractions using archived human specimens. Four different methods were tested on seven different archived tissues: (A) Qiagen QIAamp DNA FFPE kit for DNA (QD), (B) TRIzol DNA/RNA extraction method for DNA and RNA (TRI), (C) Qiagen AllPrep DNA/RNA FFPE kit for DNA and RNA (QDR), and (D) Ambion RecoverAll™ Total Nucleic Acid Isolation (AMB) for DNA and for RNA. Each nucleic acid extraction was done in triplicate to determine technical reproducibility.

**Figure 3:**
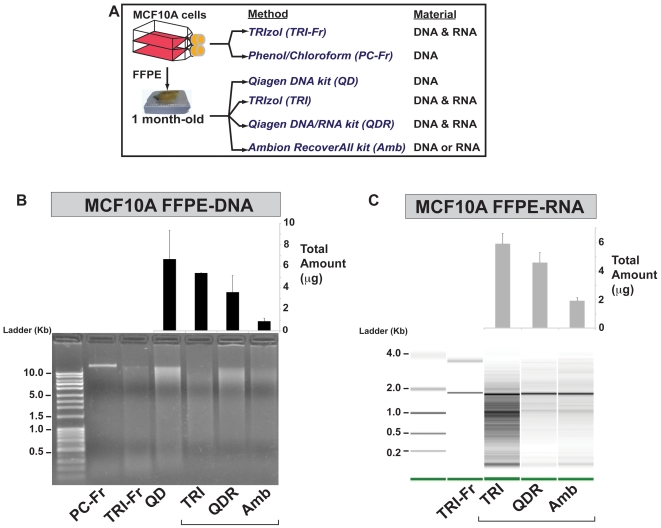
Summary of sequential recovery of DNA and RNA from MCF10A Fresh and FFPE samples using different extraction methods. (A) Schematic representation of cell culture and DNA/RNA extraction methods used with matched fresh and 1 month-old formalin-fixed paraffin-embedded (FFPE) human mammary epithelial MCF10A cells. FFPE DNA and RNA extractions (QD, TRI, QDR, AMB) were performed in triplicate using three 10 µm sections for each replicate. (B) Analysis of RNA extracted from matched fresh and FFPE MCF10A cells. Total RNA extracted from fresh cells using TRIzol (TRI-Fr; Lane 2), and total RNA extracted from FFPE cells using TRIzol (TRI; lane 3), Qiagen QIAamp DNA/RNA extraction kit (QDR; lane 4), and AMBion RecoverAll™ Total Nucleic Acid Isolation kit (AMB; lane 5) was analyzed and quantified using an Agilent 2100 Bioanalyzer 6000 Nanochip (size ladder in lane 1). The bar graph placed above the Bioanalyzer image displays total amounts of RNA recovered from three consecutive 10 µm sections, in triplicate experiments, using the three different methods (TRI, QDR, AMB). (C) Analysis of genomic DNA extracted from matched fresh and FFPE MCF10A cells. DNA was extracted from fresh cells using a phenol/chloroform based method (PC-Fr; lane 2), and TRIzol (TRI-Fr lane 3); and from FFPE cells using Qiagen QIAamp DNA FFPE kit (QD; lane 4), TRIzol DNA/RNA extraction method (TRI; lane 5), Qiagen AllPrep DNA/RNA FFPE kit (QDR; lane 6), and AMBion RecoverAll™ Total Nucleic Acid Isolation kit (AMB; lane 7) was analyzed on a 1% agarose gel (size ladder in lane 1). The bar graph placed above the agarose gel displays total amounts of DNA recovered alone (QD), simultaneously with RNA (TRI, QDR), or separately from RNA (AMB), using three consecutive 10 µm sections, in triplicate experiments for each method.

We then sought to compare efficiency of our **TRI**zol-based FFPE DNA/RNA co-extraction method (**TRI**; Fig. 2B) to that of two types of commercially available methods, the **Q**iagen AllPrep **D**NA/**R**NA FFPE kit (**QDR**; Fig. 2C), for co-extraction of DNA and RNA, and the **Amb**ion RecoverAll™ Total Nucleic Acid Isolation kit (**AMB**; Fig. 2D), for separate recovery of DNA and RNA, performed by splitting the pk-digested FFPE tissue. We used the **Q**iagen QIAamp **D**NA FFPE kit as a control for sole recovery of FFPE-DNA (Fig. 2A, **QD**) and tested the four different extractions with seven different archived tissues (Fig. 2, muscle, liver, heart, lung, thyroid, kidney, and breast). We observed that sole recovery of FFPE-DNA (Fig. 2A) only provided higher yields than the two co-extraction methods (TRI, QDR) for the liver tissue, but systematically higher yields than AMB. The QDR showed slightly higher FFPE-DNA recovery than our method (TRI) for all tissues but breast (Fig. 2B and 2C). As expected, both co-extraction approaches provided much higher FFPE-DNA yields than AMB (compare Fig. 2B and 2C to 2D). For FFPE-RNA recovery, our optimized approach (TRI) systematically provided much higher yields than QDR and AMB (Fig. 2B and 2C). Considering that we split the pK-digested tissue in two tubes for AMB, to allow recovery of FFPE-DNA and –RNA, we also compared our method (TRI) and AMB for extraction of FFPE-RNA alone (FFPE-DNA could still be recovered with our method as it is obtained from the lower aqueous phase of TRIzol) and determined that our approach still consistently provided higher RNA yields (Figure S2.). Then, using matched fresh and one-month old FFPE MCF10A cells (Fig. 3A) we explored the efficiency and reproducibility of the different methods. In this controlled experiment, we noted that the extraction of FFPE-DNA alone, using QD, yielded the highest amounts of genomic DNA from FFPE cells (Fig. 3B). For these experiments, TRI provided both the highest FFPE-DNA and FFPE-RNA yields when compared to the QDR and AMB methods (Fig. 3B and 3C). We then analyzed and compared the quality of the genomic DNA recovered from matched fresh and FFPE MCF10A cells on an agarose gel (Fig. 3B, gel). To obtain the highest DNA quality from fresh cells, we used a phenol-chloroform (PC-Fr) approach, and observed that DNA recovered from fresh cells, using TRIzol, displayed a mild profile of degradation (Fig. 3B, gel). For genomic DNA recovered from FFPE cells, QD and QDR appeared to provide higher molecular weight products than TRI and AMB (Fig. 3B, gel). These experiments demonstrate that medium to high quality genomic DNA was recovered from the 1 month-old FFPE MCF10A specimen. Generally, older archived specimens yield lower quality genomic DNA (Figure S3.). We note that when comparing the performance of the two co-extraction methods (TRI and QDR), when using older archived specimens, our optimized approach provided on average twice the amount of genomic DNA obtained with QDR (Figure S3.). We also analyzed the RNA obtained from matched fresh and 1 month-old FFPE cells, on an Agilent Bioanalyzer, and observed that TRI, QDR and AMB provided similar medium to low quality material with observable 18S ribosomal RNA (Fig. 3C, gel).

### Micro-RNA Expression Profiling of Matched Fresh and FFPE RNA

**Figure 4:**
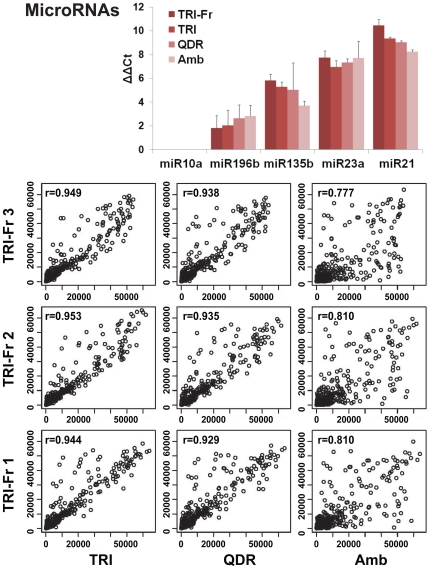
MicroRNA expression analysis of matched fresh and FFPE RNA from MCF10A cells using different RNA extraction methods. The upper panel displays a graphic representation of quantitative RT-PCR (Taqman® miRNA assays). Measurements obtain for miR-10a, miR-196b, miR-135b, miR-32a and miR-21 using matched fresh and FFPE RNA from MCF10A cells. MiRNAs were quantified using FFPE RNA extracted with TRIzol (TRI), Qiagen AllPrep DNA/RNA FFPE (QDR), AMBion RecoverAll™ Total Nucleic Acid Isolation (AMB) kits and compared to control RNA extracted from fresh cells with TRIzol (TRI-Fr). Results are represented as ΔδC_t_ (δC_t_ target miRNA - δC_t_ miR-10a (least expressed miRNA)). The lower panels show the comparison of global miRNA quantification obtained between fresh and FFPE RNA samples using the Illumina miRNA platform. Comparisons were performed between triplicate RNA extractions obtained from matched fresh (TRI-Fr1, TRI-Fr2, TRI-Fr3) and FFPE (TRI1-3, QDR1-3, and AMB1-3) cells. The correlation coefficient (r) between matched fresh and FFPE RNAs is displayed in each graph.

Firstly, we sought to determine if the different FFPE-DNA/RNA extraction methods might influence miRNA expression measures. Using five miRNAs with known differential expression (from low to high) in fresh MCF10A cells (Fig. 4 bar graph, see TRI-Fr), we performed qRT-PCR with FFPE-RNA recovered by TRI, QDR, and AMB (Fig. 4 bar graph). QRT-PCR data for miR-135b and miR-21 showed significant decreases, and for miR-196b a significant increase in expression for FFPE-RNA recovered by QDR and AMB, compared to the expression levels of miRNAs recovered from fresh cells (Fig. 4, bar graph TRI-Fr). We then sought to determine if the extraction method might influence global expression profiling and used the Illumina miRNA expression profiling platform to compare expression of 1,146 miRNAs between matched fresh and FFPE-RNA recovered by TRI, QDR, AMB, each in triplicate measures (Fig. 4, microarray data panels). Our results indicate that miRNAs measured in FFPE-RNA recovered by TRI have the highest correlation with fresh RNA (*r*> = 0.944), when compared with QDR (*r*> = 0.929) and AMB (*r*> = 0.810). We observed that AMB provided FFPE-RNA where miRNAs had the lowest correlation with fresh RNA, further validating the qRT-PCR results and indicating that this method of extraction appears to be the least suited for microRNA recovery from FFPE cells.

### Gene Expression Analysis of Matched Fresh and FFPE RNA

**Figure 5:**
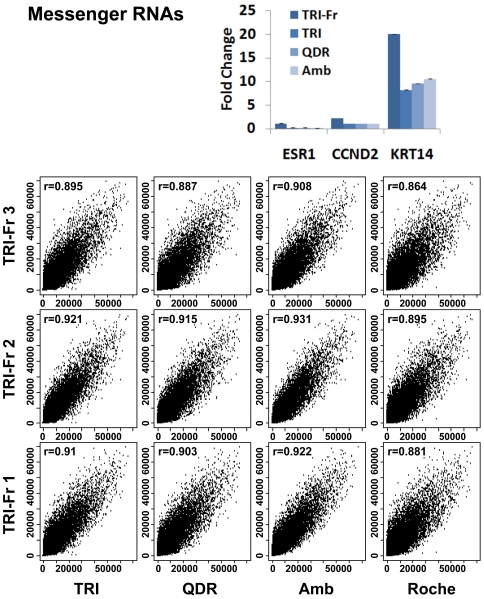
Messenger RNA expression analysis of matched fresh and FFPE RNA using different RNA extraction methods. The upper panel displays a graphic representation of quantitative RT-PCR (Taqman® mRNA assays) Measurements obtained for ESR1, CCND2 and KRT14 genes using matched fresh and FFPE RNA from MCF10A cells. The three genes were quantified using matched fresh RNA recovered with TRIzol (TRI-Fr), and FFPE RNA recovered with TRIzol (TRI), with Qiagen AllPrep DNA/RNA FFPE (QDR), with AMBion RecoverAll™ Total Nucleic Acid Isolation (AMB) and with the Roche RNA FFPE (Roche) kits. The results are represented as fold changes. The lower panels show the comparison of global mRNA quantifications obtained between fresh and FFPE RNA samples using the Illumina whole-Genome DASL platform. The different panels display comparison between triplicate RNA extractions from matched fresh (TRI-Fr1, TRI-Fr2, TRI-Fr3 (bottom to top panel)) and FFPE (TRI1-3, QDR1-3, AMB1-3 and Roche1-3 (from left to right panel)) cells. The correlation coefficient (r) between matched fresh and FFPE RNAs is displayed in each graph.

Next, we sought to determine if mRNA expression might be influenced by the FFPE-RNA extraction method and compared matched fresh and FFPE-RNA recovered by TRI, QDR, and AMB. First, we performed qRT-PCR experiments on three differentially expressed genes in fresh MCF10A cells, ESR1 for low (MCF10A cells are considered ER negative cells), CCND2 for intermediate, and KRT14 for high expression (Fig. 5, bar graph). Our data show that Taqman® qRT-PCR primers detect a significant decrease in expression in FFPE-RNA (Fig. 5, bar graph see three shades of blue), when compared to matched fresh RNA (Fig. 5, bar graph see dark blue). Next, we used the Illumina whole-genome cDNA-mediated Annealing, Selection, Extension and Ligation (WG-DASL) assay for high-throughput expression profiling of 24,526 genes to compare matched fresh and FFPE-RNA. Following Illumina's instructions for analysis of FFPE-RNA with the WG-DASL assay, we used FFPE-RNA recovered with the high-pure RNA paraffin kit from Roche (Fig. 5, microarray data panels Roche). Based on gene expression analyses, we observed a high correlation between matched fresh and FFPE-RNA (*r*> = 0.881), with greater correlation using FFPE-RNA obtained with AMB (*r*> = 0.908) and TRI (*r*> = 0.895). We noted that primers used for the WG-DASL assay span 50 nucleotides, whereas primers used to quantify ESR1, CCND2 and KRT14 spanned 62, 64 and 69 nucleotides, respectively, which might account for the decrease in expression measured in FFPE-RNA, when compared with fresh RNA.

### Methylation Analysis of Genomic DNA from Matched Fresh and FFPE DNA

**Figure 6:**
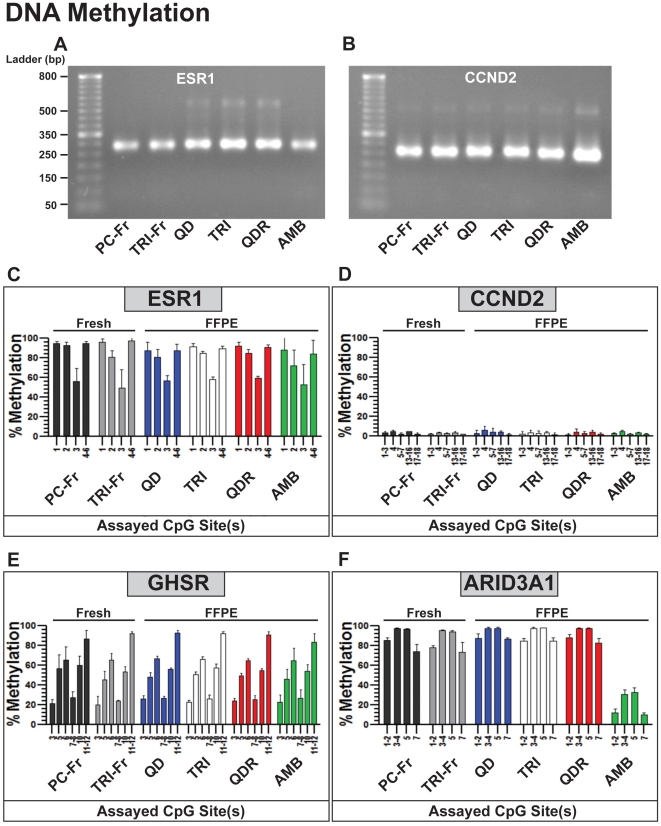
Methylation analysis of CpG regions in genes of interest using matched fresh and FFPE genomic DNA obtained by different extraction methods. Representative 2% agarose gel electrophoresis images of PCR products for (A) ESR1 and (B) CCND2 genes. Graphs depict methylation values as a percentage for CpG dinucleotide rich regions in (C) ESR1, (D) CCND2, (E) GHSR, and (F) ARID3A as assayed via the MassARRAY system (Sequenom). Data were analyzed and confirmed using the MassArray R script statistical package. Methylation values for fresh MCF10A DNA isolated with control methods (DNA from fresh cells recovered by phenol/chloroform (PC-Fr) and from FFPE cells using the Qiagen QIAamp DNA FFPE kit (QD)) are compared against methods used for matched FFPE DNA (TRIzol extraction (TRI), Qiagen AllPrep DNA/RNA FFPE (QDR), and AMBion RecoverAll™ Total Nucleic Acid Isolation (AMB)). The bar graphs display the correlation between DNA methylation measurements obtained from fresh genomic DNA and each FFPE genomic DNA recovered by the different extraction methods.

Finally, we chose to perform single methylation assays to assess the quality of genomic FFPE-DNA, by comparing it to matched fresh genomic DNA. Our approach combines PCR, a nucleotide sensitive reaction (Fig. 6A–B, ESR1 and CCND2), and mass spectrometry (MassARRAY EpiTYPER), a state-of-the-art analytical technology for measuring atomic mass differences (Fig. 6C–F). Using bisulfite-converted DNA from matched fresh and FFPE cells, we quantified methylated CpG islands in the promoter regions of *ESR1* and *GHSR*, in intron 1 of *CCND2*, and in intron 3 of *ARID3A1* (Fig. 6C–F), identified using the MassArray Statistical package [27]. Our results show that the methylation patterns of the regions analyzed for *ESR1* and *CCND2* correlate with the qRT-PCR data measured in Figure 5 (high expression/low methylation and low expression/high methylation near). Our results also show that methylation patterns observed in fresh DNA, purified by phenol-chloroform (Fig. 6C–F, PC-Fr) and by TRIzol (Fig. 6C–F, TRI-Fr), are well reproducible in FFPE-DNA obtained with the different approaches (Fig. 6C–F, QD, TRI, QDR, AMB). The PCR products obtained for the CpG islands tested for *ESR1* and *CCND2* were of comparable sizes between matched fresh and FFPE-DNAs (Fig. 6A–B) providing identical methylation patterns between fresh and FFPE-DNAs for each gene and for each extraction approach (Fig. 6C–D). We also tested *GHSR* and *ARID3A1*, two genes non-expressed in MCF10A cells (Fig. 6E–F) and only observed methylation differences with AMB, with a 60–80% decrease for *ARID3A1*. Our results show that only AMB provides FFPE-DNA that displays high variability in methylation patterns of the CpG islands measured.

## Discussion

In this study, we optimized a TRIzol-based (Invitrogen, CA, USA) approach for co-extraction of genomic DNA and total RNA from archived specimens within a single reaction [11]. Our approach allows maximal co-extraction of both nucleic acids without having to split the proteinase-K digested FFPE tissue prior to nucleic acid recovery or having to use additional FFPE tissue to obtain sufficient amounts of nucleic acid material. When compared to two commercial kits (Qiagen All-prep DNA/RNA FFPE kit and Ambion RecoverAll™ Total Nucleic acid isolation kit), in the context of matched fresh and FFPE MCF10A breast cells, we observed that our approach provided higher FFPE-DNA and –RNA yields as well as higher quality material for throughput analyses of mRNA, miRNA, and methylation analysis of genomic DNA.

For FFPE-RNA recovery, we showed that our extraction method (TRI) is superior to the two commercial kits (QDR, AMB). Considering that RNA is highly degradable in solution and that some studies suggested that mildly degraded FFPE-RNA could still be subjected to linear amplification and conventional microarray analyses [12], FFPE-RNA extraction methods, based on proteinase-K (pK) digestion, have been extensively shortened (15 minutes at 56°C for QDR, 15 minutes at 50°C for AMB, compared to 45 minutes at 59°C for TRI) to improve RNA quality. In particular the AMB, which in its earlier version suggested a 2–3 h digest at 55°C [33] has been shortened to a 15 minutes digest at 55°C, a modification that might affect FFPE-RNA yields. Considering that the whole-genome cDNA mediated selection extension ligation (WG-DASL) assay, from Illumina, is designed to interrogate 50 nucleotide regions by RT and PCR [10], [26], [33], and massively-parallel sequencing technologies is designed for the analysis of short RNA sequences (reads<100 bps) [13], and in light of recent studies demonstrating that longer pK digestion provide larger amounts of FFPE-RNA and better analytical data [34], [35], the need for short pK digestion and recovery of high quality RNA has decreased. In fact, using the WG-DASL, our mRNA expression profiling data shows the high correlation between matched fresh and FFPE-RNA obtained by all methods regardless of pK digest durations, with AMB providing the highest correlation (*r*> = 0.908). However, for miRNA expression analysis, we observed that AMB provided the lowest correlation ratios (*r*> = 0.810), between fresh and FFPE-RNA, when compared to the QDR (*r*> = 0.929) and TRI (*r*> = 0.944), suggesting that different FFPE-RNA extraction methods can quantitatively and qualitatively affect the analysis of miRNAs. However, our analyses reveal that co-extraction of DNA and RNA does not affect miRNA expression profiling results demonstrating that this approach provides high quality mRNA and miRNAs for molecular analyses.

For extraction of FFPE-DNA, we selected the Qiagen QiaAmp FFPE DNA kit (QD; Qiagen, CA, USA), as a control for yield and quality, because it has been shown to be a robust approach when compared to other methods and kits [36] and the recovered FFPE-DNA has successfully been used for genotyping studies [37], array CGH [38], genome-wide massively-parallel sequencing [13], and methylation studies [39]. Our results show that QD provides larger amounts of FFPE-DNA than co-extraction (TRI, QDR) or separate extraction (AMB) methods. Based on our analyses of freshly fixed specimens (1 month-old FFPE MCF10A), however, both co-extraction methods (TRI, QDR) still provide high DNA yields when compared to QD (80–90% of FFPE-DNA recovered by QD). When testing older archived specimens, which provide genomic DNA of lower quality (Figure S3.), our analysis revealed that TRI performed better than QDR. These experiments suggest that while being time consuming (45 min pK for RNA, 48 hours pK digest, and use of the QD kit for DNA extraction) TRI is an efficient co-extraction approach for recovery of genomic DNA from older archived specimens. For analysis of the FFPE-DNA from 1 month-old archived MCF10 cells, we used bisulfite conversion and PCR reactions to assay hypo- and hyper-methylated CpG islands of FFPE genomic DNA. Our analyses of four different CpG islands for four different genes suggests that TRI and QDR provide higher quality FFPE-DNA than AMB, which yielded material that displayed higher variation in methylation levels. TRI, which incorporates the use of the QD kit for FFPE-DNA purification, and QDR include a heat-treatment step at 90°c to increase FFPE-DNA quality through removal of FFPE-DNA/protein cross-links [20]–[23]. This heat-treatment step, which is not described in the procedure of AMB, might account for the discrepancies, between matched fresh and FFPE-DNA, observed in our methylation analysis data. It is important to note that when using older FFPE specimens, which yield lower quality genomic DNA, methylation analyses should be performed on CpG islands spanning less than 300 bp for consistent results (Figure S3.)

Our analyses demonstrated that the two co-extraction methods tested (optimized TRIzol method (TRI), and Qiagen AllPrep DNA/RNA FFPE kit (QDR)) provided higher yields as well as more reliable material for molecular studies than the separate extraction method (Ambion RecoverAll™ kit (AMB)). However, advantages and disadvantages of either method should be weighted carefully. On one side, the QDR has a short pK digestion (15 minutes), and might be automated, but it might not provide the highest amounts of FFPE DNA and RNA. On the other side our optimized method (TRI) requires two digests (45 min and 48 h), use the QD kit for final purification, and is incompatible with automation (due to the use of TRIzol), but our results indicate that it provides higher genomic DNA yields when used with older archived specimens (Figure S3.) and generally higher RNA yields than QDR. For large-scale studies, automation might be important, and thus the method described by Hennig *et al.* [2010], in which nucleic acids released by proteinase K digestion are magnetically purified, split, and subjected to RNAse for DNA purification and DNAse for RNA purification, might be more appropriate [40]. However, our results demonstrate that while the use of nucleases (RNAse or DNAse) assures higher DNA or RNA quality, dividing the pK-digested tissue solution in smaller fractions significantly decreases the amount of DNA and RNA recoverable from a single sample and thus represents a limiting approach for correlative studies or storage of optimal amounts of material for future studies. It is important to note that several other commercial kits commercialized for FFPE-DNA and –RNA recovery only allow separate extraction by splitting the pk-digested solution and include: the Norgen FFPE RNA/DNA kit (Norgen Biotek, Canada); the AxyPrep Mag FFPE (DNA/RNA/miRNA) kit (Axygen Biosciences, CA, USA); and the Aline® FFPE Magapure kit (Aline Bioscience, MA, USA).

In conclusion, our study is the first to demonstrate that high-quality FFPE-DNA can be purified from the lower aqueous phase of TRIzol, without affecting optimal recovery of FFPE-RNA from the upper organic phase. We demonstrated that co-extraction of DNA and RNA from a single archived specimen is highly efficient and provides the options of direct usage or storage of material for additional or subsequent studies. Based on our experiments we advise researchers to extract genomic DNA and total RNA at the same time for effective use of archived specimens.

## Supporting Information

Figure S1 **Co-extraction of total RNA and genomic DNA from fresh mouse tissues using TRIzol.** RNA and DNA were extracted from brain, muscle, heart and liver in triplicate to determine technical reproducibility using TRIzol and following manufacturer's instructions. Based on simultaneous extractions of DNA and RNA performed using TRIzol we consistently recovered more RNA than DNA and recovery of DNA appears highly reproducible.
(TIF)

Figure S2 **Comparison of RNA extraction between the Ambion RecoverAll™ kit (AMB) and the TRIzol-based optimized method (TRI) using archived normal and tumor human breast tissues.** Total RNA was recovered from normal breast tissue (see left side of graph) and from tumor breast tissue (see right side of graph). For normal tissue (left side of graph), total RNA was extracted from two, three, and four 10 µm sections, in triplicate experiments, using either the AMB or TRI methods. For tumor tissue (left right of graph), total RNA was extracted from one, two, and three 10 µm sections, in triplicate experiments, using either the AMB or TRI methods. The average of total RNA, in micrograms, of three experiments was plotted and error bars were determined for each individual condition.
(TIF)

Figure S3 **Electrophoretic and methylation analyses of genomic DNA recovered from older formalin-fixed paraffin-embedded benign breast disease tissue specimens.** A. Analysis of 200 ng of genomic DNA recovered from 8, 13, 20, 27 and 31 year-old BBD tissue specimens using the TRIzol-based optimized method (TRI) and the Qiagen AllPrep DNA/RNA FFPE (QDR) kit. For each specimen 5× 10 µm sections were used for each method and the total amounts of genomic DNA recovered are displayed below the image of the agarose gel, showing that TRI provides at least twice the amount of DNA than QDR. The genomic DNA displays an overall degraded profile identical in both methods. B. 1% agarose gel analysis of 25 (low quality DNA) and 27 (medium quality DNA) year-old breast specimens displaying significant differences in genomic DNA quality. C. and D. Methylation analyses of CpG regions of ESR1 (283 bp region) and CCND2 (261 bp region) using FFPE genomic DNA from the 25 and 27 year-old BBD tissue specimens and representative images of the PCR products on a 2% agarose gel, respectively. Lower quality genomic DNA (25 year old specimen) did not yield a PCR product indicating either absence of methylation or failed PCR reaction, possibly due to low genomic DNA quality.
(TIF)
