# Supplementary figures and images for: Effective DNA/RNA Co-Extraction for Analysis of MicroRNAs, mRNAs, and Genomic DNA from Formalin-Fixed Paraffin-Embedded Specimens
Source: PLoS One. 2012 Apr 13;7(4):e34683. doi: 10.1371/journal.pone.0034683 (PMC3326040; doi:10.1371/journal.pone.0034683)

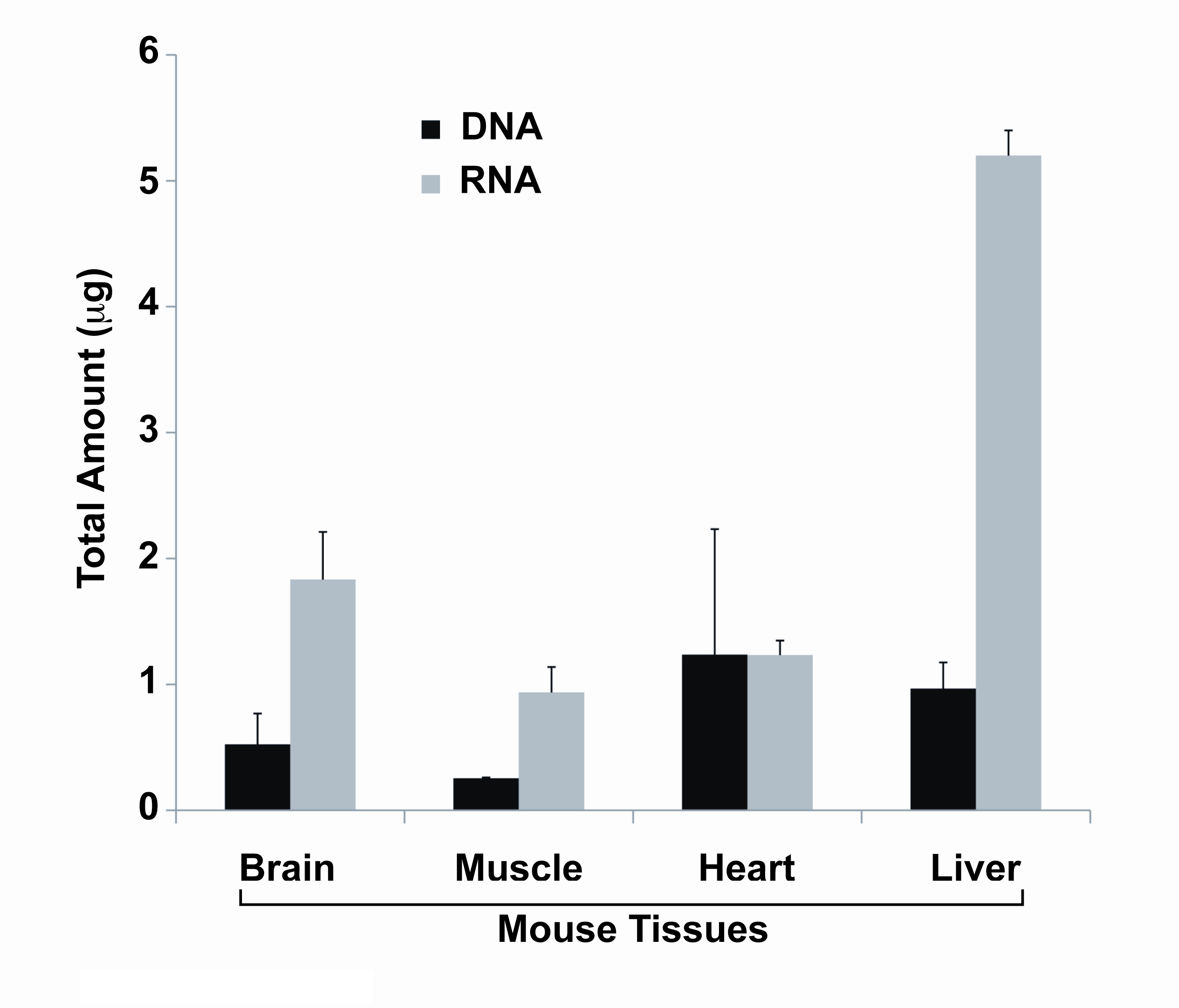

Supplement: Figure S1 — Co-extraction of total RNA and genomic DNA from fresh mouse tissues using TRIzol. RNA and DNA were extracted from brain, muscle, heart and liver in triplicate to determine technical reproducibility using TRIzol and following manufacturer's instructions. Based on simultaneous extractions of DNA and RNA performed using TRIzol we consistently recovered more RNA than DNA and recovery of DNA appears highly reproducible. (TIF) [file pone.0034683.s001.tif]

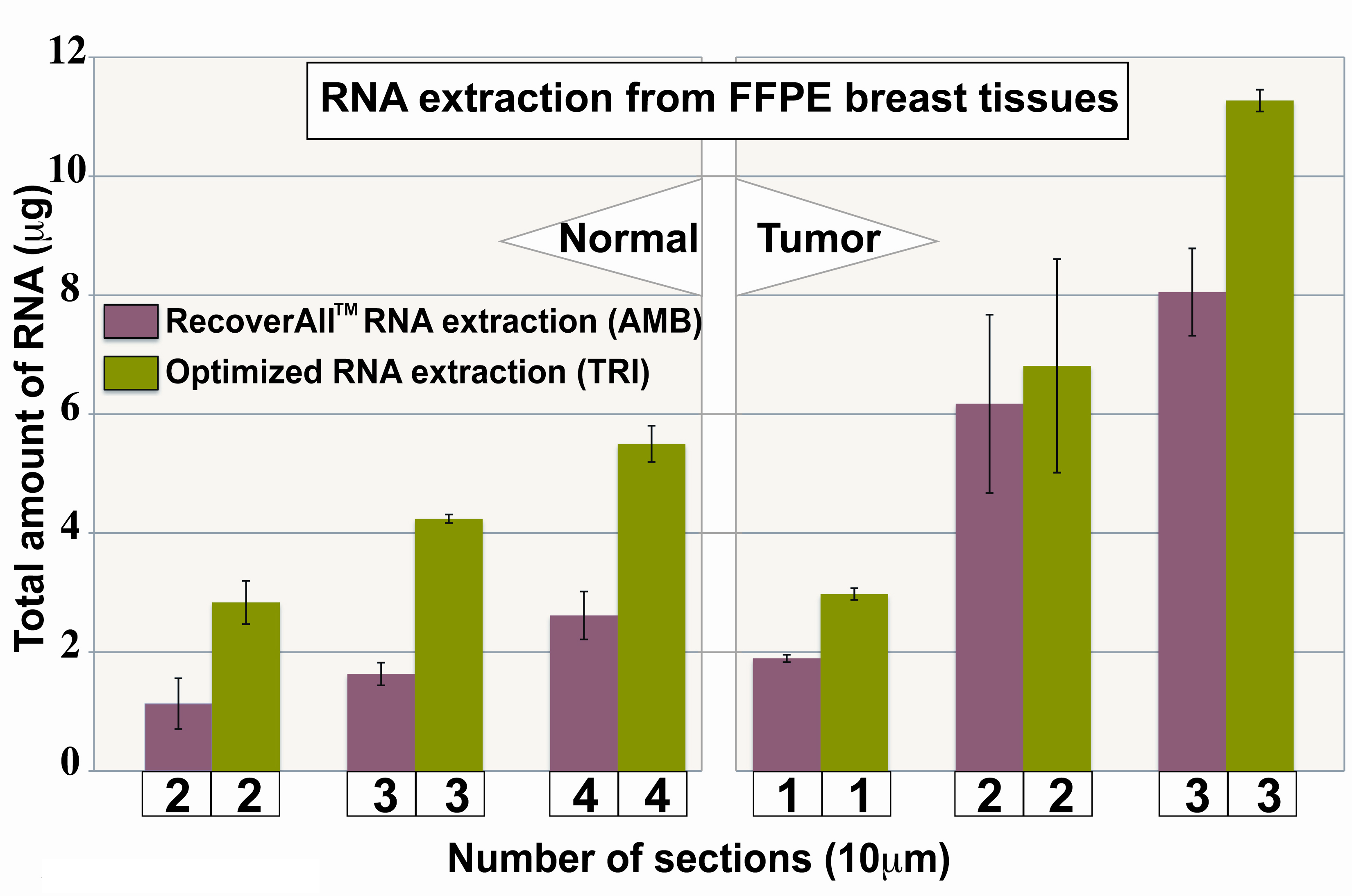

Supplement: Figure S2 — Comparison of RNA extraction between the Ambion RecoverAll™ kit (AMB) and the TRIzol-based optimized method (TRI) using archived normal and tumor human breast tissues. Total RNA was recovered from normal breast tissue (see left side of graph) and from tumor breast tissue (see right side of graph). For normal tissue (left side of graph), total RNA was extracted from two, three, and four 10 µm sections, in triplicate experiments, using either the AMB or TRI methods. For tumor tissue (left right of graph), total RNA was extracted from one, two, and three 10 µm sections, in triplicate experiments, using either the AMB or TRI methods. The average of total RNA, in micrograms, of three experiments was plotted and error bars were determined for each individual condition. (TIF) [file pone.0034683.s002.tif]

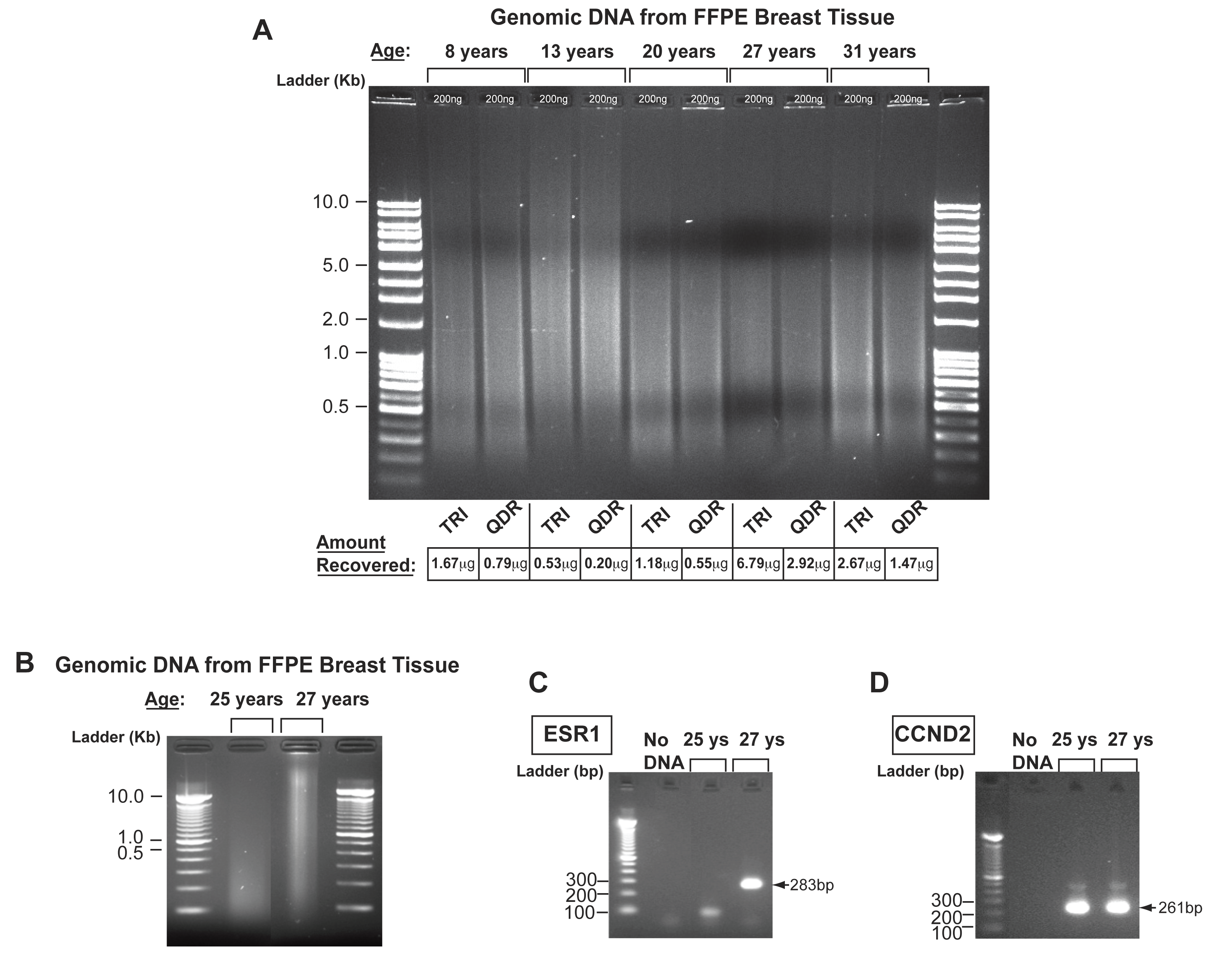

Supplement: Figure S3 — Electrophoretic and methylation analyses of genomic DNA recovered from older formalin-fixed paraffin-embedded benign breast disease tissue specimens. A. Analysis of 200 ng of genomic DNA recovered from 8, 13, 20, 27 and 31 year-old BBD tissue specimens using the TRIzol-based optimized method (TRI) and the Qiagen AllPrep DNA/RNA FFPE (QDR) kit. For each specimen 5× 10 µm sections were used for each method and the total amounts of genomic DNA recovered are displayed below the image of the agarose gel, showing that TRI provides at least twice the amount of DNA than QDR. The genomic DNA displays an overall degraded profile identical in both methods. B. 1% agarose gel analysis of 25 (low quality DNA) and 27 (medium quality DNA) year-old breast specimens displaying significant differences in genomic DNA quality. C. and D. Methylation analyses of CpG regions of ESR1 (283 bp region) and CCND2 (261 bp region) using FFPE genomic DNA from the 25 and 27 year-old BBD tissue specimens and representative images of the PCR products on a 2% agarose gel, respectively. Lower quality genomic DNA (25 year old specimen) did not yield a PCR product indicating either absence of methylation or failed PCR reaction, possibly due to low genomic DNA quality. (TIF) [file pone.0034683.s003.tif]
